# The dietary patterns derived by reduced-rank regression in association with Framingham risk score and lower DASH score in Hoveyzeh cohort study

**DOI:** 10.1038/s41598-023-37809-3

**Published:** 2023-07-08

**Authors:** Marzieh Shoja, Fatemeh Borazjani, Kambiz Ahmadi Angali, Seyed Ahmad Hosseini, Seyed Jalal Hashemi

**Affiliations:** 1 Nutrition and Metabolic Diseases Research Center and clinical sciences research institute, Ahvaz Jundishapur University of Medical Sciences, Ahvaz, Iran; 2 Department of Biostatistics, School of Health Sciences, Ahvaz Jundishapur University of Medical Sciences, Ahvaz, Iran; 3 Alimentary Tract Research Center, Ahvaz Jundishapur University of Medical Sciences, Ahvaz, Iran; 4 Department of Nutrition, Faculty of Allied Sciences, Ahvaz Jundishapur University of Medical Sciences, Ahvaz, Iran

**Keywords:** Diseases, Health care, Medical research, Risk factors

## Abstract

The relationship between dietary patterns (DPs) and cardiovascular disease (CVD) has been the subject of much research, but given the significance of this disease, studying the factors affecting it through different methodological considerations is of utmost importance. This study aimed to investigate the association between the four dietary patterns (DPs) derived from reduced-rank regression (RRR) and the risk of CVD predicted by the Framingham Risk Score (FRS) in the Arab residence of Khuzestan, Iran. Furthermore, the predefined Dietary Approaches to Stop Hypertension (DASH) would be used as a comparative model to assess the validity of the extracted DPs. In this cross-sectional study, 5799 individuals aged 35–70 without a CVD diagnosis were selected among the participants of the Hoveyzeh cohort study (HCS). The Risk of CVD was assessed using the FRS model. A semi-quantitative food frequency questionnaire evaluated dietary intake. Four DPs were derived using RRR with 28 food groups as predictors and total protein (g/d), fiber(g/d), fat(g/d), and magnesium intake (mg/d) as response variables. Multinomial and binary logistic regression were used to assess the relationship of DPs with intermediate (10–20%) and high (> 20%) levels of FRS and lower DASH scores (< 4.5), respectively. Four primary DPs were derived, which explained 89.10 of the total explained variance in participants’ dietary intake. Multinomial regression was applied between FRS (10–20%) and (> 20%) across quartiles of four identified DPs**.** After adjustment for potential confounders, higher tendency to 1st and 2nd DPs in Model 1, OR = 4.67 (95% CI 3.65; 6.01), OR = 1.42 (95% CI 1.13; 1.79) were presented accordingly. The 1st DP, characterized by higher intake of refined grains and lower intake of vegetables oil, sugar, mayonnaise and artificial juices, the 2nd DP characterized by higher intake of hydrogenated fat and lower consumption of tomato sauce and soft drink was associated with greater odds of CVD with the intermediate level of FRS. However, higher adherence to the 3rd DP, characterized by higher intake of fruits, vegetables and legumes and lower intake of fish, egg, red meat, processed meat, mayonnaise, sugar and artificial juices, the 4th DP characterized by higher intake of coffee, nuts and lower intake of sugar, mayonnaise and artificial juices was associated with a lower risk of FRS. Moreover, lower DASH score considered in binary logistic regression across quartiles of four identified dietary patterns. 1st and 2nd DPs were directly related to lower DASH scores, while 3rd and 4th DPs had high comparability with the DASH diet and inversely contributed to the lower DASH score. Total DASH score was significantly correlated to four derived DPs. Our findings confirm the current knowledge regarding the beneficial effects of healthy plant-based DPs and the avoidance of high-fat and processed foods to prevent CVD.

## Introduction

Cardiovascular diseases (CVD) are described as a cluster of ischemic heart disease, stroke, heart failure, a peripheral arterial disease that affects the heart and blood vessels^1,2^. CVD remains the prominent cause of mortality globally, accounting for one of every three deaths worldwide^3^. Reports from Iran suggest that about 46% of all deaths could be attributed to CVD^4^.

Several factors have been identified to impact the onset and severity of CVD, among which smoking status, levels of cholesterol and high-density lipoproteins (HDL), systolic blood pressure, and weight status are of significant importance^5^. These parameters, along with age and sex, are used as input variables to the Framingham Risk Score (FRS) model, which is a simplified screening tool used to predict the risk of CVD within 10 years^6^. Applying lifestyle changes to modify the said factors significantly prevented or improved the course of CVD^5^. Dietary changes can have a significant positive impact on health and can reverse the progression of cardiovascular disease^7^.

Studying DPs is superior to individual foods or nutrients as it better captures the complexities of the eating habits of a population and recognizes the synergistic or antagonistic effects that foods have on each other^8^. Reduced rank regression (RRR) is a tool used to identify DPs in a set of pre-defined response variables that are hypothesized to affect the disease outcome based on previous evidence. By combining hypothesis-driven and exploratory data-driven approaches, RRR stands out as an ideal method to derive DPs that predict disease outcomes and provide better insight into the diet-disease pathway^9^.

Evidence abounds regarding the complex association of dietary fat with the risk of CVD^10,11^. However, too much fat in the diet, regardless of the type, can raise cholesterol levels, leading to plaque formation and atherosclerosis^11,12^. Another controversial macronutrient, protein, whose effects on heart health are not yet wholly understood, has been the subject of many studies^13,14^. In a most recent one, it was reported that the elevated amino acid levels in the blood due to high-protein diets might cause plaque formation by affecting macrophage signaling pathways^15^. In addition, fiber has been shown to considerably decrease CVD risk markers such as weight, low density lipoprotein (LDL) cholesterol, and blood glucose level^16,17^. Its beneficial effects on reducing blood pressure and inflammation were also noted^18^. It is necessary to say that with increasing prevalence of hypertension in Iran^19^, we have no specific dietary guidelines to evaluate dietary patterns and their contribution with hypertension or CVD.

Moreover, a meta-analysis of more than 1 million individuals found that dietary magnesium intake is associated with a reduced risk of stroke, heart failure, diabetes, and all-cause mortality^20^. Therefore, we chose these nutrients as our response variables in the RRR analysis to identify posterior DPs that are meaningful for the outcome of CVD.

The dietary approaches to stop hypertension (DASH) developed by the National Institutes of Health as the “best overall” diet^21^ and as an effective interventional strategy to prevent and manage CVD^22–24^ and CVD risk factors^25^. The basic characteristics of DASH diet are focused on consumption of foods rich in potassium, calcium, magnesium, protein, and fiber^26^. Moreover, it is relatively more compatible with the Iranian dietary habits compared to other healthy predefined dietary patterns.

Given that, DASH diet has been recommended as a first-line nonpharmacological therapy along with lifestyle modifications for the treatment of many chronic diseases^25,27^.

Hence, the DASH score will be used as a comparative model to assess the validity of the DPs identified in this study to evaluate its relationship with the CVD risk factors among the participants. Generally, whether the derived DPs are accordance with it since the DASH diet is very well described to have favorable impact on blood pressure control^25^.

Previous studies investigating the association of DPs using RRR with different CVD risk factors were primarily conducted in North America and Europe. These studies focused on pathways based on disease-related inflammatory biomarkers^28^, serum lipids^29^, and nutrient intakes^30^. Eating habits hugely vary from middle-east to west, but few studies on middle-eastern populations are available. Furthermore, no study has considered fat, protein, fiber, and magnesium intake as response variables. Thus, using RRR, we aimed to identify DPs that are exclusively associated with the intake of the said nutrients and subsequently examine the associations between these posteriori DPs and the risk of CVD predicted by FRS and compare them with the priori dietary pattern, DASH dietary score.

## Methods

### Study design

This cross-sectional study was conducted on data from the first phase of the national Persian Cohort, Hoveyzeh cohort study (HCS). The HCS is a prospective Arab population-based study on non-communicable diseases in which 10,009 adults aged 35–70 from May 2016 to August 2018 in Hoveyzeh and Rofayyeh cities, Khuzestan province; Southwest Iran participated.

The HCS cohort study is one of the components of the Prospective Epidemiological Research Studies in Iran (the PERSIAN Cohort Study) approved by the Ethics Committees in the Ministry of Health and Medical Education, the Digestive Diseases Research Institute, Tehran University of Medical Sciences, Iran. Details of this study had been published elsewhere^31,32^.

**Figure 1:**
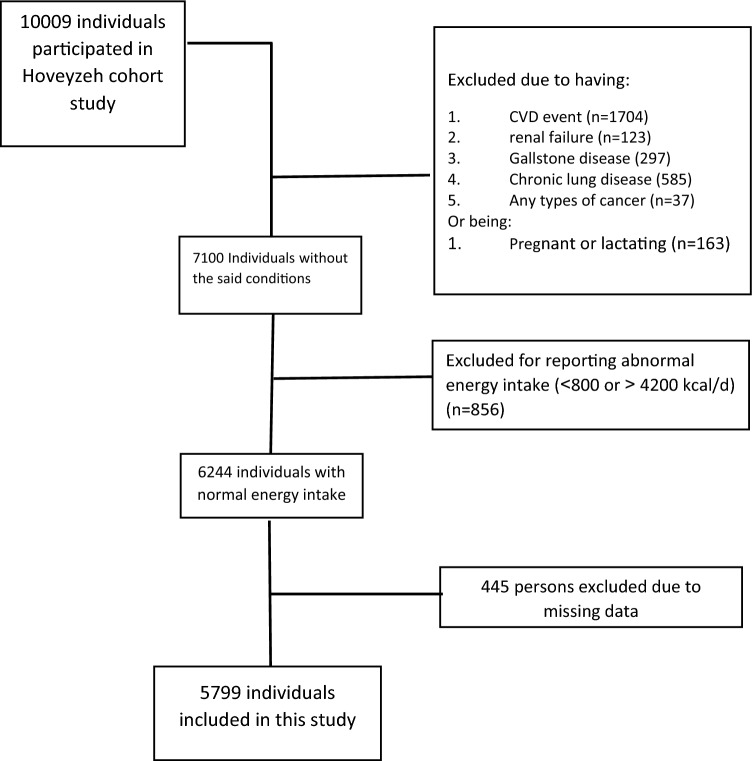
Study flow chart.

For the present study, patients with renal failure, chronic lung disease, CVD event, Gallstone disease, any types of cancer, and pregnant and lactating women were excluded (n = 2909). Also, we considered energy intake ranging from 800 to 4200 kcal/day as usual^33^. In accordance, participants whose energy intake fell outside this range were omitted (n = 856). Additionally, 445 participants with missing data were excluded from the current study. Overall, the data from 5799 participants were applied to the study (Fig. 1).

The study was approved by the Ethics Committee of Ahvaz Jundishapur university of medical sciences (No: IR.AJUMS.REC.1399.739).

### Anthropometric measurements

Weight was assessed using digital scales (Seca 755) to the nearest 100 g, while the individuals wore light garments and no shoes. Height was assessed to the nearest 0.5 cm by a Seca stadiometer (Seca company. Model 206. Germany) in a standing position with shoulders in a normal alignment and without shoes. Body mass index (BMI) was calculated by dividing weight (kg) by height squared (m^2^). Waist circumference (WC) was measured to the nearest 0.1 cm at the level of the umbilicus over light clothing, at the end of normal expiration, and without putting any pressure on the body surface using a non-flexible tape meter. Hip circumference was assessed at the maximal protrusion of the gluteal muscles. By dividing WC (cm) to hip circumference (cm), the waist to hip ratio (WHR) was calculated.

### Biochemical indices and blood pressure assessment

After 10–12 h of fasting, blood samples were collected from each participant. To separate serum from blood, samples were centrifuged for 15 min at 1000 rpm and stored at − 80 °C until analysis. Further details of the procedures are provided in the prior study^32^. Fasting blood glucose (FBG) was measured by the glucose oxidase method. Total cholesterol (TC) and HDL were determined by enzymatic kits (Pars Azmoon, Iran). Systolic and diastolic blood pressure was measured twice from each arm by Riester sphygmomanometers, with an interval of 10 mins. The mean of the two was reported.

### Dietary assessment

A semi-quantitative food frequency questionnaire (FFQ) containing 130 food items, was used to assess the usual food intakes of individuals^32^. Participants were asked to report the frequency of intake of each item during the past year on a daily, weekly, monthly, or annual basis. A trained dietitian fulfilled all questionnaires through face-to-face interviews with participants. The amount of each food was converted to gram/day using household measures. The actual food intake (g/day) was then transferred to Nutritionist IV to calculate the total energy and nutrients intake.

### DP determination

DPs were derived using RRR, a relatively new statistical technique to derive DPs that maximize the variation explained by response variables, chosen based on their relation to the outcome of the disease. For the current analysis, protein (g/d), fat (g/d), fiber (g/d), and magnesium (mg/d) were selected as response variables, which were previously shown to be related to the pathophysiology of CVD^34–37^. Twenty-eight food groups were used as predictor variables in this study (Table 1). Intakes (g/d) of all 28 food groups and the four response variables were used to derive four DPs. A food groups with factor loading equal and over 0.11 was used to describe the characteristics of the dietary pattern^38^.

**Table 1:** Food groupings and their components.

Food group	Dietary components
Processed meat	Sausage- hamburger- sausages,
Red meat	All kinds of red meats, heart, liver, kidney, trip and rennet, brain, tongue, head sheep meat, leg sheep meat
Fish	Fish
Poultry	Chicken
Egg	Egg
Dairy	Milk, yogurt, cheese, chocolate milk, cream, ice cream
Tea	Tea
Coffee	Coffee
Artificial juice	Packed juices
Fruits	Cantaloupe, watermelon, melon, apple, orange, apricot, cherry, fig, peach, pear, citrus fruits, date, kiwi, grapes, pomegranate, strawberry, banana, grapefruit, other fruits, dried figs, dried dates, and other dried fruit
Tomato	Tomato, tomato paste
Tomato sauce	Tomato sauce
Vegetables	Carrot, spinach, lettuce, mixed vegetables, eggplant, zucchini, pumpkin, local vegetables, pepper, mushroom, cucumber, garlic, cabbages, onion, and other vegetables
Potato	Potato
Legumes	Bean, chickpea, split pea, soybean, lentil, kidney bean
Whole grain	Barbari*, Sangak*, Taftoon*, Barley
Refine grain	Lavash*, baguette, rice, macaroni, pasta
Snack	Biscuits, corn chips, potato chips
Pickle	Pickles, salted vegetables
Nuts	Peanut, almond, walnut, pistachio, hazelnut, roasted seeds
Sweet dessert	Cakes, cookies, chocolate, pastry, honey, jam, halva
Sesame paste	sesame paste
Sugar	Sugar, sugar cube, candy, sugar candy
Hydrogenated fat	Solid oil, margarine, Solid vegetable oil
Butter	Butter
Non-alcoholic drinks (soft drink)	Low-alcohol beer
vegetables oil	Liquid oil and olive oil
Mayonnaise	Mayonnaise

*A kind of traditional bread.

### DASH score

The DASH score was determined for each participant using the Mellen et al. formula^39^. It takes into account the intake of 9 nutrients; protein, total fat, saturated fat, cholesterol, fiber, magnesium, calcium, potassium, and sodium. They were standardized to calorie intake (except for macronutrients). A score of 1 was given to the said nutrients if the DASH target level was met, a score of 0.5 if the intermediate target was met, and a score of 0 if neither target was met. Individual nutrient scores were then pooled together to calculate a total DASH score ranging from zero to nine; 9 represents full adherence to the diet. In the binary analysis conducted in the present study, commitment to the DASH diet was determined if a score of ≥ 4.5 was gained. A score less than 4.5 was considered as negligible adherence.

### FRS score

FRS was used to assess the risk of CVD, based on the six coronary risk factors including age (years old), gender (male/female), TC (mg/dl), HDL-cholesterol (mg/dl), systolic blood pressure (mm Hg), and smoking habits (Yes/ No). The following cutoffs were considered to calculate the FRS: TC < 160, 160–199, 200–239, 240–279 and  ≥ 280 mg/dL; for systolic blood pressure: < 120, 120–129, 130–139, 140–159, and ≥ 160 mmHg; and for HDL-C: < 40, 40–49, 50–59, and ≥ 60 mg/dL. Ten year risk in percentage was calculated by total points(1 point 6%, 2 points 8%, 3 points 10%, 4 points 12%, 5 points 16%, 6 points 20%, 7 points 25%, 10 points or more > 30%)^40^.

Based on the total score, CVD risk percentage over 10 years was categorized into three groups include of; low risk (< 10%), intermediate risk (10–20%) and high risk (> 20%) of contracting CVD within 10 years^40^.

### Statistical analysis

**Figure 2:**
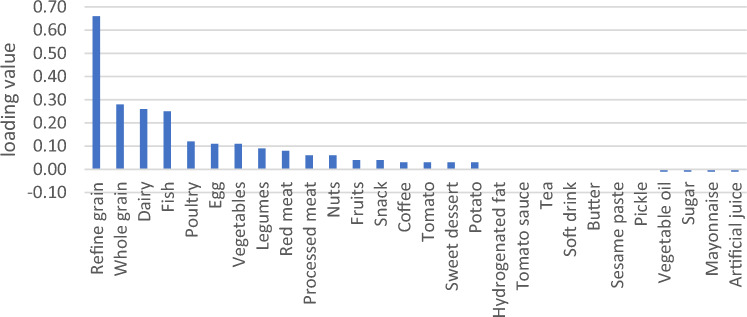
Factor loading of food groups in four identified DPs. DP1 food groups.

**Figure 3:**
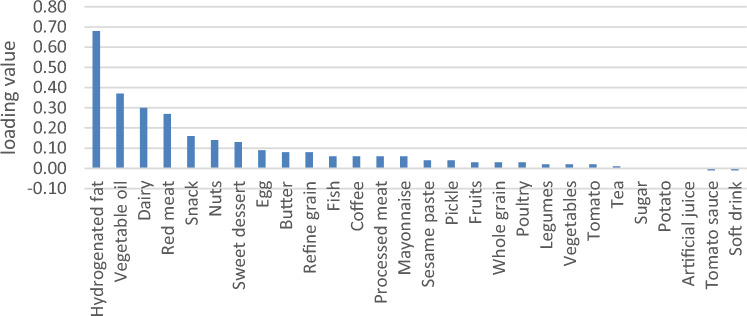
Factor loading of food groups in four identified DPs. DP2 food groups.

**Figure 4:**
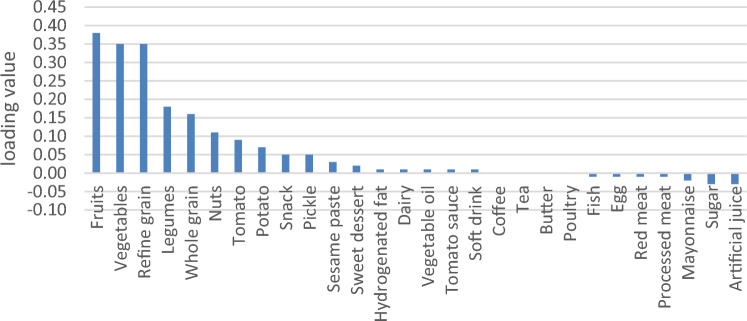
Factor loading of food groups in four identified DPs. DP3 food groups.

**Figure 5:**
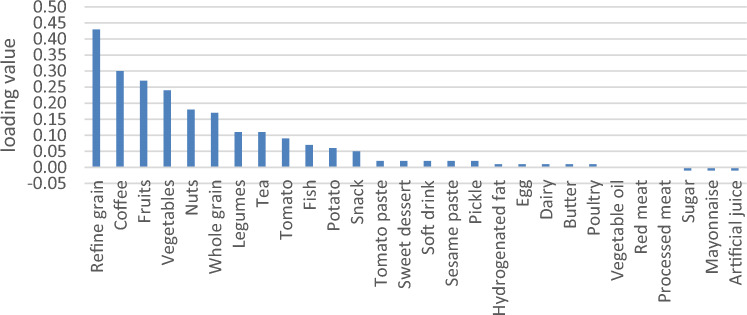
Factor loading of food groups in four identified DPs. DP4 food groups.

General characteristics were expressed as means ± SDs for continuous variables and numbers and percentages for categorical variables (Table 2). We used RRR to extract four primary DPs based on response variables (Table 3). Higher rates of explained variance indicate a stronger association. Factor loadings were also reported (Figs. 2, 3, 4 and 5). A more positive loading means a robust direct association between the food group and the pattern. Correlation between response variables and RRR-derived dietary patterns were presented in Supplementary Table 1. The association between dietary patterns and response variables were shown in Supplementary Table 2. The Pearson correlation between food groups and response variables were expressed in Supplementary Table 3. DASH score was calculated in accordance with the Mellen et al. formula^39^. Hence, the nine nutrients include of; saturated fatty acid (g/d), total fat (g/d), total protein (g/d), cholesterol (g/d), fiber (g/d), magnesium (mg/d), calcium (mg/d), potassium (mg/d) and sodium (mg/d) were considered to find out the DASH score. Moreover, maximum score of 9 was accounted by summation of all nutrients that met the target level score, intermediate target level counted ≥ 4.5 and less than intermediate level does not meet the DASH target level. It is need to be mentioned that some nutrients presented as percentage from total daily energy consumption and the rest of nutrients accounted per 1000 kcal of energy intakes, were expressed in Supplementary Table 4.

**Table 2:** Basic characteristics of studied participants.

Characteristics	
Age, year, mean(SD)	47.36 (8.86)
Sex, male %	35.9
Education %	
Under diploma	92.0
Academic	8.0
Wealth score category %	
Poorest	20.1
Poor	20.9
Moderate	20.0
Rich	19.8
Richest	19.2
Marital status %	
Single	4.4
Married	87.3
Widow	6.2
Divorced	2.0
Anthropometric indices, mean(SD)	
Weight, kg	76.62 (15.32)
BMI, kg/m^2^	28.51 (5.32)
WHR	0.94 (0.06)
Biochemical indices, mean(SD)	
FBG, mg/dl	112.71 (50.08)
TG, mg/dl	161.36 (104.89)
TC, mg/dl	189.27 (40.63)
HDL, mg/dl	50.42 (12.06)
Blood pressure, mm Hg, mean(SD)	
DBP	71.17 (11.18)
SBP	112.83 (18.42)
Drinking alcohol %	1.8
Smoking %	17.6
Physical activity, (MET hour/week)	37.32 (5.37)
Diabetic %	22.6
Hypertension %	18.0
Fatty live %	6.9
Dietary intake, mean (SD)	
Total energy (kcal/d)	2898.71 (693.21)
Total fat (g/d)	63.38 (23.24)
Carbohydrates (g/d)	501.10 (127.55)
Fiber (g/d)	31.56 (9.16)
Protein (g/d)	90.17 (22.92)
Framingham risk score%	
Low: < 10%	92.7
Intermediate: 10–20%	6.7
High: > 20%	0.7
DASH score, mean (SD)	2.93 (0.92)

*Mean (SD).

*BMI* body mass index, *WHR* waist to hip ratio, *FBS* fasting blood sugar, *TC* total cholesterol, *HDL* high density lipoprotein, *DBP* diastolic blood pressure, *SBP* systolic blood pressure, *DASH* dietary approaches to stop hypertension.

**Table 3:** Explained variance of response variables.

Food pattern	Protein	Fat	Fiber	Magnesium	Current	Total
1st DP	75.06	46.82	65.35	82.99	67.56	67.56
2nd DP	76.36	92.70	71.66	86.15	14.16	81.72
3rd DP	91.35	93.02	78.28	87.10	5.72	87.44
4th DP	91.56	93.04	80.66	91.07	1.66	89.10

Pearson correlation was applied to see the association between DASH score as priori dietary pattern and response variables using to derive RRR based dietary pattern considered as posteriori dietary pattern, presented in Supplementary Table 5.

Participants were then categorized into quartiles of DPs score. Multinomial logistic regression was used to estimate the odds ratios (ORs) and 95% confidence intervals (CIs) for FRS across quartiles of DP scores in different models. To study the similarity between the DPs and the DASH diet, good adherence to DASH was used as a reference. In contrast, poor adherence to DASH diet considered as lower DASH score was compared across quartiles of DPs via Binary logistic regression in crude and adjusted models. Age, BMI, energy intake which is indicated as model 1, smoking, sex, wealth score and physical activity, in addition to model1 is regarded as model2 that were controlled in the adjusted models. SAS statistical software (SAS Studio 3.71; SAS Institute Inc.) to identify the posteriori dietary patterns and SPSS version 22 were applied to determine priori dietary pattern and perform the analyses. P < 0.05 was considered to be statistically significant.

### Ethics approval and consent to participate

This project was approved by the research council (research project number: NRC_9905) and the ethics committee (research ethics number: IR.AJUMS.REC.1399.739) of Ahvaz Jundishapur University of medical sciences. Participants were fully informed about the study objectives and methods, and were assured of the confidentiality of their information. An informed consent was obtained from all subjects. All methods were carried out in accordance with relevant guidelines and regulations.

## Results

Basic characteristics of the participants are displayed in Table 2. With average age of 47.36 years, only 8% have academic achievement and more than 80% are married. Participants were nearly equivalent across all levels of wealth score, with impaired fasting blood glucose and borderline levels of TG and TC. Overall, the mean DASH score was reported to be 2.93, although 92% of them were classified as low risk with FRS less than 10%.

Four primary DPs were derived are shown in Table 3, which explained 89.10% of the total explained variance in participants’ dietary intake. Of Four DPs were identified; the 1st DP explained 67.56% of response variation and 9.07% of those in food intake; therefore, it was most commonly practiced in our population. It consisted of staples such as refined grains, whole grains, fish, poultry, egg and a lower intake of vegetables oil, sugar, mayonnaise and artificial juice. The 2nd DP explained 14.16% of response variation and 3.6% of those in food intake; composed of hydrogenated oil, red meat, dairy, snacks, vegetable oil, sweet desserts and a lower intake of sugar, mayonnaise and artificial juice. 2nd DP displayed a high negative correlation with fiber, that indicate a lower consumption in this dietary pattern (Supplementary Table 1). The 3rd DP, which explained 5.72% of response variation and 3.79% of those in food intake; it was loaded with fruits, vegetables, legumes and reduced intake of fish, egg, red meat, processed meat, sugar, mayonnaise and artificial juices. 3^rd^ DP showed a high negative correlation with protein, which suggests a lower protein intake in this dietary pattern (Supplementary Table 1). The 4th DP explained 1.66% of response variation and 3.73% of food intake, with high negative correlation with magnesium as response variable (Supplementary Table 1). It comprised nearly the same food groups with lower factor loadings than the 3rd dietary pattern, and besides with higher loading score of coffee, nuts and lower intake of tomato sauce and soft drink (Figs. 2, 3, 4 and 5). Compared to other patterns, the first DP had a more explained variance. Hence, it was recognized as the dominant pattern. Noteworthy, in the association between dietary patterns and response variables, the selected response variables caused total explained variance as much as 89.10% for all identified dietary patterns. In the correlation between response variables and posteriori dietary patterns, 1st DP positively was correlated with all response variables. 2nd DP highly and positively only correlated with total fat as response variables. However, 3rd DP and 4th DP negatively only related to total protein and magnesium as response variables respectively.

The association between Framingham risk score across quartiles of four identified DPs are presented in Table 4. Multivariable-adjusted odds ratios (OR) and 95% confidence intervals (CI) for FRS across quartiles of 1st DP showed that higher adherence to 1st DP in the first adjusted model was associated with greater odds of CVD risk in the intermediate level of FRS 10–20% (OR = 4.67; 95% CI 3.65, 6.01; P_trend_ < 0.001) and in the high level of FRS > 20% (OR = 2.39; 95% CI 1.19, 4.77; P_trend_ < 0.001) accordingly. In addition, higher adherence to 2nd DP in the first adjusted model was associated with higher risk of CVD in the intermediate level of FRS 10–20% (OR = 1.42; 95% CI 1.13, 1.79; P_trend_ = 0.003) but the relationship wasn’t significant when FRS > 20% was evaluated (OR = 0.83; 95% CI 0.40, 1.73; P_trend_ = 0.20). However, higher adherence to the 3rd DP in the first adjusted model was associated with lower risk of CVD in the intermediate level of FRS 10–20% (OR = 0.58; 95% CI 0.44, 0.75; P_trend_ < 0.001) and in the high level of FRS > 20% (OR = 0.47; 95% CI 0.23, 0.98; P_trend_ < 0.001) as such. Finally, 4th DP in both adjusted models were also inversely associated with FRS > 20% (OR = 0.35; 95% CI 0.13, 0.90; P_trend_ = 0.031), (OR = 0.11; 95% CI 0.02, 0.47; P_trend_ = 0.003) while the association was only significant in the second adjusted model, when FRS 10–20% was assessed (OR = 0.46; 95% CI 0.24, 0.87; P_trend_ = 0.018).

**Table 4:** Multinomial regression logistic odds ratios and 95% confidence intervals for Framingham risk score across quartiles of four identified DPs.

Variables			Q1 (n = 1366)	Q2 (n = 1426)	Q3 (n = 1479)	Q4 (n = 1528)	P-trend
				OR (95% CI)	OR (95% CI)	OR (95% CI)	
1st DP	FRS (10–20%)	Crude	1	1.43 (1.12, 1.82)	1.89 (1.50, 2.38)	2.08 (1.66, 2.62)	< 0.001
		Adjusted 1*	1	1.97 (1.52, 2.55)	3.27 (2.35, 4.21)	4.67 (3.65, 6.01)	< 0.001
		Adjusted 2**	1	0.97 (0.56, 1.68)	1.95 (1.07, 3.56)	1.93 (0.96, 3.89)	0.064
	FRS (> 20%)	Crude	1	1.28 (0.66, 2.47)	0.98 (0.48, 1.99)	1.26 (0.64, 2.45)	< 0.001
		Adjusted 1	1	1.66 (0.85, 3.23)	1.53 (0.74, 3.15)	2.39 (1.19, 4.77)	< 0.001
		Adjusted 2	1	1.21 (0.39,3.69)	0.93 (0.23, 3.70)	1.61 (0.34, 7.61)	0.543
2nd DP	FRS (10–20%)	Crude	1	1.16 (0.93, 1.45)	1.24 (1.00, 1.55)	1.31 (1.06, 1.63)	0.013
		Adjusted 1	1	1.27 (1.00, 1.60)	1.38 (1.10, 1.75)	1.42 (1.13, 1.79)	0.003
		Adjusted 2	1	1.24 (0.79, 1.94)	1.17 (0.74, 1.85)	0.95 (0.60, 1.51)	0.844
	FRS (> 20%)	Crude	1	1.37 (0.73, 2.57)	1.01 (0.51, 1.99)	0.78 (0.37, 1.61)	0.13
		Adjusted 1	1	1.47 (0.78, 2.77)	1.10 (0.56, 2.18)	0.83 (0.40, 1.73)	0.20
		Adjusted 2	1	2.26 (0.77, 6.65)	1.95 (0.64, 5.91)	1.19 (0.37, 3.81)	0.766
3rd DP	FRS (10–20%)	Crude	1	0.87 (0.70, 1.07)	0.83 (0.67, 1.03)	0.82 (0.67, 1.02)	0.10
		Adjusted 1	1	0.78 (0.57, 0.92)	0.70 (0.55, 0.90)	0.58 (0.44, 0.75)	0.001
		Adjusted 2	1	1.77 (1.08, 2.89)	1.04 (0.60, 1.80)	1.25 (0.69, 2.29)	0.452
	FRS (> 20%)	Crude	1	0.64 (0.34, 1.18)	0.41 (0,20, 0.83)	0.60 (0.32, 1.12)	0.101
		Adjusted 1	1	0.58 (0.30, 1.09)	0.37 (0.17, 0.79)	0.47 (0.23, 0.98)	0.001
		Adjusted 2	1	1.85 (0.64, 5.37)	0.78 (0.21, 2.81)	0.90 (0.22, 3.61)	0.884
4th DP	FRS (10–20%)	Crude	1	1.02 (0.82, 1.27)	1.06 (0.85, 1.31)	1.18 (0.95, 1.46)	0.363
		Adjusted 1	1	0.95 (0.74, 1.22)	0.80 (0.60, 1.07)	0.81 (0.58, 1.12)	0.409
		Adjusted 2	1	0.75 (0.45, 1.27)	0.41 (0.22, 0.74)	0.46 (0.24, 0.87)	0.018
	FRS (> 20%)	Crude	1	0.75 (0.40, 1.38)	0.58 (0.30, 1.13)	0.58 (0.30, 1.14)	0.363
		Adjusted 1	1	0.65 (0.33, 1.28)	0.41 (0.18, 0.92)	0.35 (0.13, 0.90)	0.031
		Adjusted 2	1	0.64 (0.22, 1.78)	0.20 (0.05, 0.72)	0.11 (0.02, 0.47)	0.003

*Adjusted1 for age, BMI, and energy intake.

**Adjusted for age, BMI, energy intake, sex, smoking, wealth score and physical activity.

Multivariable-adjusted OR and 95% CIs for lower DASH scores across quartiles of DPs are presented in Table 5. Higher adherence to 1st DP in the second adjusted model (OR = 10.99; 95% CI 4.70, 25.64; P < 0.0001) was associated with having higher risk of lower DASH score, while 2nd DP did not remain significant in the crude and both adjusted models. On the other hand, 3rd DP in the second quartile of crude model (OR = 0.72; 95% CI 0.44, 1.16; P P < 0.001) and 4th DP in the third quartile of crude model (OR = 0.36; 95% CI 0.21, 0.61; P = P < 0.001) were remarkably comparable to the DASH diet. Furthermore, total DASH score highly correlated with magnesium and negatively correlated with total fat intake, nutrients considered as response variables.

**Table 5:** Binary logistic regression odds ratios and 95% confidence intervals for lower DASH scores across quartiles of four identified dietary patterns.

Variables		Q1 (n = 1366)	Q2 (n = 1426)	Q3 (n = 1479)	Q4 (n = 1528)	P-trend
			OR (95% CI)	OR (95% CI)	OR (95% CI)	
1st DP	Crude	1	1.73 (1.27, 2.37)	2.63 (1.84, 374)	5.85 (3.62, 9.46)	< 0.001
	Adjusted 1*	1	1.71 (1.25, 2.34)	2.56 (1.79, 3.66)	5.67 (3.50, 9.20)	< 0.001
	Adjusted 2**	1	2.41 (1.48, 3.92)	2.92 (1.65, 5.17)	10.99 (4.70, 25.64)	0.0001
2nd DP	Crude	1	1.30 (0.92, 1.83)	1.32 (0.93,1.87)	1.57 (1.09,2.26)	0.093
	Adjusted 1	1	1.31 (0.92, 1.85)	1.31 (0.92, 1.86)	1.54 (1.07, 2.22)	0.108
	Adjusted 2	1	1.20 (0.74, 1.95)	1.32 (0.79, 2.21)	1.12 (0.65, 1.91)	0.680
3rd DP	Crude	1	0.72 (0.44, 1.16)	0.52 (0.33, 0.82)	0.23 (0.15, 0.35)	< 0.001
	Adjusted 1	1	0.67 (0.41, 1.09)	0.47 (0.29, 0.74)	0.21 (0.14, 0.32)	< 0.001
	Adjusted 2	1	0.45 (0.20, 1.01)	0.09 (0.04, 0.20)	0.01 (0.00, 0.03)	0.0001
4th DP	Crude	1	0.72 (1.40, 1.26)	0.36 (0.21, 0.61)	0.13 (0.08, 0.21)	< 0.001
	Adjusted1	1	0.41 (0.23, 0.75)	0.14 (0.08, 0.25)	0.03 (0.02, 0.05)	< 0.001
	Adjusted 2	1	0.36 (0.17, 0.73)	0.09 (0.04, 0.19)	0.03 (0.01, 0.07)	0.0001

*Adjusted for age, BMI, and energy intake.

**Adjusted for age, BMI, energy intake, sex, smoking, wealth score and physical activity.

## Discussion

### Main outcomes

The association between DPs derived from RRR and the risk of CVD predicted by FRS was investigated in the current study. After adjustment for potential confounders, we found that higher adherence to 1stDP (characterized by a higher intake of refined grains, whole grain, egg, fish and poultry) and 2nd DP (defined by red meat, snack, sweet dessert, hydrogenated fat, vegetable oil and dairy) were associated with a greater FRS in the highest quartile in contrast to lowest. Whereas participants in the highest quartile of 3rd and 4th DPs (characterized by a higher intake of fruits, vegetables, legumes, coffee and nuts) had a significantly lower level of FRS. Moreover, 3rd and 4th DPs were significantly comparable to the DASH diet, unlike 1st DP and 2nd DP.

The possible reason for present association regarding 1st and 2nd DPs and FRS, may be due to the interactions between different food groups. For instance, in 1st DP with higher intake of whole grain as anti-inflammatory properties and treatment potential for cardiovascular disease^41^ and higher consumption of fish shown to improve some cardiovascular risk factors, such as triglycerides and blood pressure^42^, although other cohort study did not reach significantly the benefit of fish intake and CVD outcomes improvement^43^. Moreover, in our study in the 1st and 2nd DPs there are unhealthy food groups including refine grain (highest loading factor in 1st DP), snack, sweet dessert and hydrogenated fat (highest loading factor in 2nd DP) that may counteract the benefit of healthy food groups.

Similarly, in a Survey conducted in Lebanon, three DPs derived by the PCA, PLS and RRR with anthropometric measurements were selected as response variables for PLS and RRR methods. PLS and RRR derived patterns explained greater variance in the outcome and were significantly associated with elevated BP. 1st and 2nd DPs (identified by whole dairy products, fried potato, refined and whole grain, sweets, cured meat, fast food sandwiches, starchy vegetables, vegetables, bottled fruit juices, meat, fish, poultry, egg, fats, olives and oils, and ice cream) from the RRR method were significantly associated with elevated BP, although the 3rd DPs (nuts and seeds, breakfast cereal, low fat dairy, hot drinks and regular soda) was inversely related. Comparable to our study, the final finding is due to existence of unhealthy and healthy food groups that hidden the favorable effect of healthy food groups^38^.

### DPs and CVD risk (Framingham risk score)

In the current study, the association between FRS and four identified DP's quartiles showed a positive significant association between 1st DP with higher refined grain loading score and 2nd DP with higher hydrogenated fat loading score contributed to higher CVD risk. Similarly, in the UK Biobank longitudinal study, DP was based on RRR considering fat nutrients as response variables and odds of increased CVD risk (Framingham risk score).

The dietary pattern characterized by higher butter and high-fat cheese was associated with higher systolic blood pressure^44^. We observed the inverse significant association between 3rd and 4th DPs which are high in (fruits, vegetables, legumes, coffee, nuts and lower sugar, artificial juices and mayonnaise) with lower odds of elevated CVD risk. In the same study from UK by higher (nuts and seeds and lower fruit and legumes intake) was associated with higher diastolic blood pressure^44^. In another prospective study TLGS from Iran DPs were identified through factor analysis and participants in the highest tertile of the healthy dietary pattern (characterized by high intake of fruits, fruit juices, vegetables, liquid oils, and nuts and lower intake of refined grains) had a lower risk of CVD development^45^. Differently, in another TLGS study, Iran, DPs expressed by PCA including healthy DP with higher factor loading for vegetables in contrast to unhealthy DP with higher factor loading for sugar sweetened beverages and more adherence to DASH, HEI were not associated with risk of hypertension^46^. TLGS study stated that energy intake increased with higher adherence to DASH diet. It is noteworthy that the favorable effect of a DASH diet on BP is due to restrict calorie intake and weight loss^47^.

Therefore, high intakes of refined grains and hydrogenated fats, along with other dietary intakes, were positively considered causative factors associated with CVD risk in this study. In comparison to the other studies; nuts, high saturated fat food components and lower refined grain plays a role in connection with a rise or decrease in CVD risk^44,45^.

Prior studies investigating this association mostly used pure data-driven patterns. Our DPs share similarities with those studies regarding the outcome and diet components. A 2nd DP known by high consumption of red meat, snacks, and desserts, was found to be associated with a higher risk of CVD in numerous studies^48,49^. Similarly, two other studies showed that the traditional Iranian diet, in which refined grain had a high factor loading (same as ours), was associated with an elevated risk of diabetes^50,51^. However, other studies found no significant association between traditional Iranian DPs and chronic diseases^52–54^. The discrepancy might be because the traditional Iranian diet is composed of both healthy (such as legumes and vegetables) and unhealthy (refined grain and red meat) food items^55^. And finally, higher consumption of several food groups, such as vegetables, fruits, and whole grains, which were commonly identified as a prudent or healthy diet, was also associated with a lower risk of CVD in previous studies^55,56^. These food groups may act through various metabolic pathways in CVD development. Based on available knowledge healthy Plant-based diets, are similar to the DASH diet rich in vegetables and whole grains and limited in refined grains, sugar-sweetened beverages, and total meat may contribute to lower BP. So, they are rich in fiber, vitamins and polyphenols, potassium and unsaturated fatty acids, and low in saturated fatty acids and sodium, to maintain healthy blood pressure (BP)^57^.

Moreover, the high amount of fiber improves blood glucose levels and lipid metabolism and decreases inflammation^58^. Whereas traditional and western diets are typically packed with high-fat processed foods and often fail to meet the recommended daily intake of the said beneficial nutrients, predisposing the body to oxidative stress and inflammation, thus paving the way for the progression of CVD^59^.

### RRR derived DPs with inclusion of different response variables in association with CVD

Several RRR studies used a variety of intermediates, including plasma lipids^29^, inflammatory markers^60^, and nutrient intakes^61^ as response variables to derive DPs predictive of the risk of CVD. Despite using different intermediates, the derived DPs shared several similarities in their food groups suggested to affect CVD. In the present study by inclusion of total protein, fat, fiber and magnesium as response variables, four DPs were derived trough RRR statistical model. Likewise, a cross-sectional study on 1026 Brazilian women used sodium, potassium, and SFA intakes as response variables and identified one pattern for further analysis that characterized by a high intake of vegetables and low intake of red meat, associated with a lower prevalence of hypertension^62^. In the association to lower risk of CVD, the 3rd and 4th DPs of current study are rich in fiber with higher intake of fruits, vegetables, legumes, nuts and coffee. Although, the 1st DP with refined grain and 2nd DPs with high fat concentration and sugary food items were related to the rise of risk. Furthermore, Livingstone et al. identified that a DP characterized by low fiber density and high sodium to potassium ratio was associated with a higher prevalence of hypertension^63^. Sun Q et al. observed that Participants with the highest score of a pattern that reflected plasma lipids and was characterized by high intakes of rice, red meat, and processed foods had a higher likelihood of developing CVD^64^. In study conducted by Johns DJ et al. among Obese Subjects used macronutrients as response variables to derive the RRR based dietary pattern characterized with chocolate, low fiber bread and other high fat food items. They reported an increase in cardiometabolic risk factors among severe obese participants, whereas one-unit increase in the DP’s score was associated with greater serum cholesterol, serum triglycerides, systolic blood pressure and diastolic blood pressure^65^.

Maddock et al. used RRR to derive a DP reflecting folate and B12 intake and homocysteine concentration characterized by high intake of vegetables and fruits and low intake of processed meat, white bread, and sugar was related to lower levels of TG and CRP^61^. Another study that used the same intermediate factors reported that a DP high in whole grain, olive oil, fresh fruit, and mushroom was related to a reduced risk of coronary heart disease^66^. In accordance, recent meta-analyses study emphasized the benefits of dietary fiber consumption for patients with CVD and hypertension^67^.As a consequence, having more tendency to the food components which is rich in fiber and lower in saturated fatty acid and sugary snack leads to reduce the risk.

### DPs and metabolic mechanism in relation to CVD

High fiber foods including whole grain and vegetables also contain other beneficial nutrients such as antioxidant, nitric oxide, leads to improve blood pressure through substantial bioavailability for use in vasodilation and decrease oxidative stress. Reducing LDL cholesterol and triglyceride may explain further benefit of fiber’s intake^68^, through improving the elasticity of blood vessel walls to reduce vascular resistance. The other related mechanism, following higher fiber intakes caused satiety and weight reduction which enhanced insulin sensitivity, may explain an important role in endothelial function and hypertension^69^.

Given that DASH diet has protective role in CVD risk reduction and has been endorsed in the USDA Dietary Guidelines for Americans, 2020–2025^70^. The DASH diet emphasizes the consumption of whole grains, fruits and vegetables, low-fat dairy, lean meat, fish, poultry, nuts, seeds, and legumes, and minimal consumption of fats and oils^71^. Therefore, DASH diet is low in sodium, saturated, trans fats and high in potassium, magnesium, calcium, nitrates, and antioxidants a combination that may contribute to cardiovascular health benefits^71^. Accumulating evidence has found a substantial role of the DASH diet, because of synergistic dietary pattern of nutrients is suggested to decreased pro-inflammatory condition, promoting endothelial function and managing CVD^72–77^.

Further strengthening the points made in this study, we discovered that the 1st and 2nd DPs were associated with poor adherence to the DASH diet. In contrast, the 3rd and 4th DPs resembled adequate adherence to the DASH diet. Participants in the 1st and 2nd DPs showed more adherence to consume refined grain and hydrogenated fat respectively. However, in the 1st DP there are healthy food groups such as whole grains and fish with higher loading values than poultry and eggs. Therefore, the combination of healthy and unhealthy food groups built this association. This came as no surprise as the 1st and 2nd DPs, contained several dietary items that are discouraged in the DASH diet (such as refined grain and hydrogenated fat) while the 3rd and 4th DPs mostly contained the items that are advocated in the DASH diet (such as fruits, vegetables, whole grains, nuts and legumes). In addition, in the present study dietary DASH score was correlated significantly to the four identified DPs.

A cross-sectional study, China Nutrition and Health Surveillance among Chine’s adult reported posterior dietary pattern extracted with selected micronutrients as response variables in RRR method and priori DASH diet-score calculated to validate the dietary pattern derived by RRR method^78^. The posterior DP was characterized by higher fresh vegetables, fruits, dairy products, less refined grains and alcohol consumption and healthy food items in Chines dietary culture, along with DASH diet presented the protective effects on both hypertension prevention and control^78^.

### Strengths and limitations

The present study used four nutrients as response variables to derive DPs from RRR and subsequently examined their associations with the risk of CVD predicted by FRS. RRR is a powerful tool for DP analysis by using response variables associated with the pathophysiology of the disease of interest. Above all, given that very few studies have used RRR to derive DPs and investigate their association with CVD in the Middle East, our study provides more scientific evidence for CVD prevention by dietary modification in the Middle-East population. However, some limitations need to be considered. First, the recall bias in reporting dietary intake is likely to affect DPs. The cross-sectional nature of our study was another limitation, as it prevented us from inferring causality. Although the nutrient-derived dietary patterns obtained using RRR with nutrients in food groups as responses explain a higher variation of response variables, it should be noted that using predictors and responses from dietary assessment tools simultaneously may violate the independence assumption for response variables. Moreover, there might have been other potential confounders that could not be measured or adjusted. Another significant limitation is the overlap between the derived DPs; as a consequence of RRR’s inability to identify uncorrelated DPs. Moreover, response target variables in RRR method, are different in other studies, so make it difficult to compare the DPs reported in other studies directly with our findings. The Arab population-based study, will reduce the generalization of findings.

## Conclusion

In summary, the findings of this study further support the knowledge regarding the benefits of DPs mainly composed of healthy plant-based foods with regards to CVD while discouraging the adoption of processed foods and red meat. However, further prospective studies are required to test the causal relationship between dietary patterns and cardiovascular health.

## Supplementary Information

Supplementary Tables.

## Data Availability

The data that support the findings of this study are available from The Hoveyzeh Cohort Study (HCS), but restrictions apply to the availability of these data, which were used under license for the current study and so are not publicly available but is available with the corresponding author upon reasonable request.

## Abbreviations

BMI: Body mass index
WC: Waist circumference
DP: Dietary pattern
RRR: Reduced-rank regression
CVD: Cardiovascular disease
DASH: Dietary approaches to stop hypertension
FRS: Framingham risk score
TC: Total cholesterol
SFA: Saturated fatty acids
HDL: High-density lipoprotein
LDL: Low-density lipoproteins
OR: Odds ratio
CI: Confidence interval
