# Supplementary material for: The dietary patterns derived by reduced-rank regression in association with Framingham risk score and lower DASH score in Hoveyzeh cohort study
Source: Sci Rep. 2023 Jul 8;13:11093. doi: 10.1038/s41598-023-37809-3 (PMC10329634; doi:10.1038/s41598-023-37809-3)
Supplement: Supplementary file 1 — Supplementary Tables. [file 41598_2023_37809_MOESM1_ESM.docx]

**Supplementary table.1**, correlation between response variables and RRR-derived dietary patterns

| Dietary patterns | Response Variables | | | |
| --- | --- | --- | --- | --- |
|  | protein | Fat | Fiber | Magnesium |
| 1^st^ DP | 0.527757 | 0.411903 | 0.49413 | 0.554656 |
| 2^nd^ DP | -0.156567 | 0.903387 | -0.323967 | -0.233292 |
| 3^rd^ DP | -0.808482 | 0.107332 | 0.539671 | 0.208786 |
| 4^th^ DP | 0.208124 | 0.052043 | 0.599697 | -0.770934 |

p-value was <0.0001

**Supplementary table2**. The association between dietary patterns and response variables

|  |  | Response Variables | | | | |  |
| --- | --- | --- | --- | --- | --- | --- | --- |
| Total explained variance | Current explained variance | magnesium | Total fiber | Total fat | Total protein | Dietary patterns | |
| 67.56 | 67.56 | 82.99 | 65.35 | 46.82 | 75.06 | 1^st^ DP | |
| 81.72 | 14.16 | 86.15 | 71.66 | 92.70 | 76.36 | 2^nd^ DP | |
| 87.44 | 5.72 | 87.10 | 78.28 | 93.02 | 91.35 | 3^rd^ DP | |
| 89.10 | 1.66 | 91.07 | 80.66 | 93.04 | 91.65 | 4^th^ DP | |

Reported as a percentage.

Conducting analysis with reduced rank regression (RRR).

**Supplementary table 3**, the Pearson correlation between food groups and response variables.

| **Food groups** | **magnesium** | **p-value** | **Total protein** | **p-value** | **Total fat** | **p-value** | **Total fiber** | **p-value** |
| --- | --- | --- | --- | --- | --- | --- | --- | --- |
| whole. Grain | 0.499 | 0.001 | 0.678 | 0.000 | 0.148 | 0.003 | 0.414 | 0.000 |
| Refine. Grain | 0.042 | 0.000 | 0.121 | 0.000 | 0.004 | 0.071 | 0.050 | 0.000 |
| Potato | 0.281 | 0.000 | 0.229 | 0.000 | 0.157 | 0.000 | 0.311 | 0.000 |
| Dairy | 0.287 | 0.000 | 0.384 | 0.000 | 0.411 | 0.000 | 0.182 | 0.000 |
| Legumes | 0.335 | 0.000 | 0.309 | 0.000 | 0.188 | 0.000 | 0.383 | 0.000 |
| red meat | 0.198 | 0.000 | 0.268 | 0.000 | 0.440 | 0.000 | 0.140 | 0.000 |
| Fruits | 0.565 | 0.000 | 0.322 | 0.000 | 0.269 | 0.000 | 0.637 | 0.000 |
| Hydrogenated fat | 0.003 | 0.825 | 0.012 | 0.363 | 0.534 | 0.000 | 0.001 | 0.967 |
| vegetable Oil | 0.144 | 0.000 | 0.071 | 0.000 | 0.104 | 0.000 | 0.162 | 0.000 |
| butter | 0.103 | 0.000 | 0.133 | 0.000 | 0.235 | 0.000 | 0.068 | 0.000 |
| Mayonnaise | 0.092 | 0.000 | 0.059 | 0.000 | 0.167 | 0.000 | 0.071 | 0.000 |
| Sweet Desert | 0.166 | 0.000 | 0.137 | 0.000 | 0.289 | 0.000 | 0.140 | 0.000 |
| Sugar | 0.078 | 0.000 | 0.078 | 0.000 | 0.089 | 0.000 | -0.017 | 0.203 |
| Pickle | 0.232 | 0.000 | 0.171 | 0.000 | 0.229 | 0.000 | 0.232 | 0.000 |
| Soft drink | 0.169 | 0.000 | 0.148 | 0.000 | 0.249 | 0.000 | 0.108 | 0.000 |
| processed meat | 0.100 | 0.000 | 0.153 | 0.000 | 0.188 | 0.000 | 0.077 | 0.000 |
| Fish | 0.288 | 0.000 | 0.393 | 0.000 | 0.205 | 0.000 | 0.192 | 0.000 |
| Poultry | 0.177 | 0.000 | 0.300 | 0.000 | 0.194 | 0.000 | 0.127 | 0.000 |
| Egg | 0.272 | 0.000 | 0.357 | 0.000 | 0.268 | 0.000 | 0.192 | 0.000 |
| Tea | 0.196 | 0.000 | 0.092 | 0.000 | 0.067 | 0.000 | 0.045 | 0.001 |
| Coffee | 0.424 | 0.000 | 0.154 | 0.000 | 0.196 | 0.000 | 0.114 | 0.000 |
| Artificial juice | 0.094 | 0.000 | 0.085 | 0.000 | 0.132 | 0.000 | 0.039 | 0.003 |
| Tomato | 0.443 | 0.000 | 0.307 | 0.000 | 0.228 | 0.000 | 0.472 | 0.000 |
| Vegetable | 0.598 | 0.000 | 0.395 | 0.000 | 0.318 | 0.000 | 0.672 | 0.000 |
| Snack | 0.232 | 0.000 | 0.128 | 0.000 | 0.322 | 0.000 | 0.192 | 0.000 |
| Nuts | 0.352 | 0.000 | 0.185 | 0.000 | 0.291 | 0.000 | 0.271 | 0.000 |
| Sesame Paste | 0.157 | 0.000 | 0.089 | 0.000 | 0.189 | 0.000 | 0.153 | 0.000 |
| Tomato sauce | 0.111 | 0.000 | 0.067 | 0.000 | 0.157 | 0.000 | 0.097 | 0.000 |

**Supplementary table 4**, the nutrients targets for DASH score.

| **Daily Nutrients** | **DASH target level** | **Score** | **DASH intermediate target level** | **Score** | **Not meet DASH target level** |
| --- | --- | --- | --- | --- | --- |
| Saturated fat (g) | ≤6% | 1 | >6-11% | 0.5 | >11% |
| Total fat (g) | ≤27% | 1 | >27-32% | 0.5 | >32% |
| Total protein (g) | ≥ 18% | 1 | <18-16.5% | 0.5 | <16.5 |
| Cholesterol (mg/1000 kcal) | ≤71.4/1000 | 1 | >71.4/1000-107.1/1000 | 0.5 | >107/1000 |
| Fiber (g/1000 kcal) | ≥14.8/1000 | 1 | <14.8/1000 – 9.5/1000 | 0.5 | <9.5/1000 |
| Magnesium (mg/1000 kcal) | ≥238/1000 | 1 | <238/1000-158/1000 | 0.5 | <158/1000 |
| Calcium (mg/1000kcal) | ≥590/1000 | 1 | <590/1000-402/1000 | 0.5 | <402/1000 |
| Potassium (mg/1000kcal) | ≥2238 /1000 | 1 | <2238/1000-1534/1000 | 0.5 | <1534/1000 |
| Sodium  (mg/1000kcal) | ≤1143/1000 | 1 | >1143/1000-1286/1000 | 0.5 | >1286/1000 |
| Total DASH score |  | 9* |  | 4.5** |  |

* Target DASH score 9. ** score 4.5 moderately meet the target.

**Supplementary table 5**, the Pearson correlation between total DASH score and four DPs.

| **Response variables** | **1^st^ DP** | **2^nd^ DP** | **3^rd^ DP** | **4^th^ DP** |
| --- | --- | --- | --- | --- |
| DASH score | 0.430 | -0.213 | 0.727 | 0.622 |
| p-value | 0.00001 | 0.00001 | 0.00001 | 0.00001 |
